# The role of attention in human motor resonance

**DOI:** 10.1371/journal.pone.0177457

**Published:** 2017-05-16

**Authors:** Guglielmo Puglisi, Antonella Leonetti, Ayelet Landau, Luca Fornia, Gabriella Cerri, Paola Borroni

**Affiliations:** 1 Department of Health Sciences, University of Milano, Medical School, Milan, Italy; 2 Department of Medical Biotechnology and Translational Medicine, University of Milano, Medical School, Milan, Italy; 3 Department of Psychology & Department of Cognitive Sciences, The Hebrew University of Jerusalem, Jerusalem, Israel; 4 Humanitas Clinical and Research Center, Rozzano, Italy; University of Bologna, ITALY

## Abstract

Observation of others' actions evokes in primary motor cortex and spinal circuits of observers a subliminal motor resonance response, which reflects the motor program encoding observed actions. We investigated the role of attention in human motor resonance with four experimental conditions, explored in different subject groups: in the first *explicit* condition, subjects were asked to observe a rhythmic hand flexion-extension movement performed live in front of them. In two other conditions subjects had to monitor the activity of a LED light mounted on the oscillating hand. The hand was clearly visible but it was not the focus of subjects’ attention: in the *semi-implicit* condition hand movement was relevant to task completion, while in the *implicit* condition it was irrelevant. In a fourth, *baseline*, condition subjects observed the rhythmic oscillation of a metal platform. Motor resonance was measured with the H-reflex technique as the excitability modulation of cortico-spinal motorneurons driving a hand flexor muscle. As expected, a normal resonant response developed in the *explicit* condition, and no resonant response in the *baseline* condition. Resonant responses also developed in both *semi-implicit* and *implicit* conditions and, surprisingly, were not different from each other, indicating that viewing an action is, *per se*, a powerful stimulus for the action observation network, even when it is not the primary focus of subjects’ attention and even when irrelevant to the task. However, the amplitude of these responses was much reduced compared to the *explicit* condition, and the phase-lock between the time courses of observed movement and resonant motor program was lost. In conclusion, different parameters of the response were differently affected by subtraction of attentional resources with respect to the *explicit* condition: time course and muscle selection were preserved while the activation of motor circuits resulted in much reduced amplitude and lost its kinematic specificity.

## Introduction

Cortical motor areas, typically responsible for programming the execution of movement, are also involved in numerous cognitive processes ranging from motor planning, estimation and prediction, to motor imagery, action perception and motor learning [1–8]. Relevant to the present paper is the important role in action perception of brain regions associated with motor functions, the fronto-parietal action observation network (AON), which are active during both the execution of movement and the observation of actions performed by others, in the latter case without producing actual movement (“motor resonance” [9,10]). In primary motor cortex (M1) several functional parameters of the motor resonant response can be identified, consistent with the different aspects comprising a motor program, i.e. the neural code normally assembled by this cortical area controlling movement execution: response amplitude (force recruitment), accuracy of temporal structure (onset of muscle contraction) and selection of muscle-specific pathways (control of joint angular position). The M1 motor resonance response replicates the structure of the motor command that the observer would utilize if executing the same movement he/she is observing [11–16].

Since observers are not aware or in control of the activation of their motor pathways during the action observation tasks, it is generally assumed that the AON is automatically recruited [17–20]. Behavioral studies have also suggested that recruitment of the AON is an automatic process, showing priming and interference effects on movement execution induced by movement observation [21–23], even when irrelevant or hindering the primary task [24,25]. The distinction between automatic and controlled processes is typically based on how these are initiated and maintained: automatic processes are generally thought to be rather inflexible and triggered involuntarily, needing little to no attentive resources, and thus occurring regardless of current available resources, while controlled processes require both attention and voluntary cognitive control. Although automatic processes can affect controlled processes, the opposite, by definition, does not occur [26,27]. However, motor resonance is a complex response and may be composed of different computational operations, not all equally demanding of the same amount of attentional resources. Thus the automatic vs controlled activation of the AON should not be posed in mutually exclusive terms; similar to other perceptual networks, the AON could be recruited by the adequate stimulus in a bottom-up manner, and still be subject to additional modulation consequent to either limitation of available neural resources or deployment of top-down influences, such as attention [28–30]. The question of whether motor resonant response is an automatic, stimulus-driven response generated each time an action is viewed by the observer [17,31] or whether it can be affected by concurrent cognitive processes occupying the observers’ attentional resources, has often been investigated indirectly, in studies manipulating the context of the action observation task [32–36] rather than the attention of observers to the task. This question has important theoretical implications for the proposed role of motor resonance in cognitive functions such as action understanding, imitation, motor learning and rehabilitation [37]. Recent studies have begun investigating the role of attention in shaping motor resonance responses. The effect on the motor resonant response of shifts of attention between externally and internally generated representations, such as in action observation *vs* motor imagery respectively, have been described in both behavioral and electrophysiological experiments [38–41]. Other neuroimaging and neurophysiological studies, have described a decrease in neural activity of cortical motor areas when action observation is disturbed by a simultaneous task requiring cognitive resources [42–45]. However, the techniques utilized (fMR, EEG and MEG), while providing important data useful to map responses in different cortical areas, are not suitable to interpret whether the remaining level of neural activity measured in motor-related cortical areas corresponds to a residual portion of the motor resonant response and, if so, what specific aspects of the response may have been disturbed by attentive interference with the action observation task. These techniques measure the activity, either excitatory or inhibitory, of large populations of cortical neurons, and do not have the adequate spatial and temporal resolution to describe activation of muscle-specific pathways in real time. Instead, in the present study we utilize a more direct neurophysiological approach, the H-reflex technique, which provides a quantitative measurement of the resonant response, directly related to the number of motorneurons in a muscle-specific pool activated during action observation, with high temporal resolution (see Methods). The advantage of the technique is that, while having the same high temporal resolution of other neurophysiological techniques, such as for example TMS, it samples the activity of muscle-specific motor circuits without magnetic (or electrical) stimulation of the cortex, providing an independent measurement of the same cortical phenomena while avoiding potential interference with cortical processing.

Motor resonance is a cortical phenomenon, but previous evidence has shown that the activation of motor circuits in M1, reflecting the subliminal motor program that codes the observed action, actually reaches and modulates the excitability of spinal motorneurons [46–48]. This evidence is supported by recent elegant studies in the macaque monkey describing the activity of corticospinal visuo-motor neurons (mirror neurons), which descend from M1 to spinal motorneurons innervating hand muscles with either monosynaptic or interneuronal connections [49–51]. The evidence from these studies implies that M1 pyramidal neurons actively fire in response to action observation, but that their modulation of the membrane potential of spinal motorneurons remains subliminal for movement execution. In the absence of actual movement, measuring variations in excitability of spinal motorneurons with the H-reflex technique amounts to measuring the activity of motor cortical output to the spinal cord, i.e. the result of the activation of these cortical areas by action observation.

Specifically, the observation of a flexion-extension movement of the wrist will be utilized to describe the excitability modulation induced in the observer’s spinal and cortical motor pathways of a wrist flexor muscle (flexor carpi radialis, FCR). This is a simple intransitive movement: motor resonant responses have been classically described in monkeys for goal directed actions, and intransitive movements have also been shown to be effective stimuli for human observers [24,52–57]. During the observation of a flexion-extension hand movement, the pattern of subliminal facilitation elicited in observers’ motor pathways reveals a time-locked activation of the same muscular groups that would be used to perform it, i.e. the motorneuronal pool activating the FCR muscle shows maximal facilitation during observed flexion and minimal during observed extension. Moreover, motorneurons controlling antagonist muscles (flexor and extensor carpi radialis) are modulated in phase opposition, reflecting their natural reciprocal activation during execution of hand oscillations [46]. Critically, since the observed flexion-extension movement has a sinusoidal time course, the same mathematical function can be utilized to fit both observed wrist oscillation and resonance effects on the observer’s wrist motor circuits and to generate a continuous parallel representation of the two events [58]. With this tool, we can explore the role of attention in the development of the motor resonant response with different experimental conditions in which the attention of subjects is diverted to different cognitive tasks, compared to a condition in which subjects are only observing the same hand movement. We hypothesize that if viewing the moving hand can automatically recruit the motor system even when the attention of observers is not specifically directed to it, the motor resonant response will develop normally. If instead attention is necessary for the development of the resonant response, manipulation of attention will perturb or even cancel the H-reflex modulation; in addition, we expect that the different parameters of the resonant response might be differently affected, depending on their relative need of attentive resources.

## Materials and methods

A total of 56 volunteers (34 females, average age 27, range 19–40) participated in an experiment with 4 different conditions; 14 different subjects were tested in each condition. The sample size was determined based on *a priori* power analysis for a one-way ANOVA with an alpha level of .05 s performed on data from a pilot study with 5 subjects for each condition (G-power 3.2 software [59]). The experimental protocol was approved by the University of Milano ethics committee and written informed consent was obtained from each subject, in compliance with the rules of the declaration of Helsinki. All subjects had normal or corrected-to-normal vision, no history of neurological disorders. All were right handed according to the standard Edinburgh Handedness Inventory [60].

### Experimental conditions

In order to evaluate the role of attention on the motor resonant response, the focus of attentive resources deployed to the observation of a hand movement was manipulated by changing its relevance. Following is a summary of the experimental conditions; details of experimental procedures are given below in the *Experimental set-up* and *Data acquisition* sections.

Four different experimental conditions were explored. In the “*explicit observation”* condition (n = 14), subjects were instructed to devote their attention to action observation; this approach replicates the experimental condition of a great number of published action observation experiments in which subjects are explicitly instructed to observe an action. In the “*semi-implicit observation”* condition (n = 14) subjects were instructed to engage their attention in a different task, but the execution of the task required the implicit observation of the same hand action as in the *explicit* condition, i.e. subjects were never instructed to observe the action performed in front of them, but needed to do so in order to complete their task. In the “*implicit observation”* condition (n = 14) subjects were instructed to perform yet a different task, which they carried out independently of the fact that the same hand action as in the previous two conditions was performed in front of them. Finally, in the *“baseline observation”* condition (n = 14) subjects were instructed to observe an inanimate object which was moved in an identical manner as the hand in the previous three experimental conditions. Based on previous results showing that observation of an inanimate object does not elicit a motor resonant response ([46]), the goal of this condition was to establish a baseline reflex amplitude variation in the absence of resonant modulation, against which to evaluate the results of the other conditions.

### Experimental set-up

The experimental set-up has been previously utilized and described [46,47]. The hand movement was performed live in front of each subject by one of the experimenters, from here on called “mover” (S1 Fig). Movers were seated in an armchair, with the right arm bent at the elbow and the hand fixed in prone position to a platform that could oscillate around the wrist axis. Observers were also seated in an armchair, about 1.5m directly facing the mover, with their right arm comfortably resting on an armrest in prone position and were instructed not to move during the experimental trials (as continuously monitored by the same electromyography (EMG) electrodes utilized to record H-reflexes, see *Data acquisition* section). The mover’s hand rested on a metal platform which moved solidly with his/her hand; the position of the platform was recorded continuously as the analogue output of a Spectrol 534 1kΩ potentiometer coaxial with its pivot, subsequently digitized at 250Hz and saved for later analysis. Thus, in all trials of all conditions, the continuous recording of the platform angular position provided a direct measurement of the actual time course of observed movements. In each trial movers performed a series of 10 flexion-extension cycles of the hand, following the 1Hz tempo of a metronome, heard through headphones. Single trials lasted about 10s, and were separated from the following trial by 20-30s intervals. A total of 100 trials were obtained in each subject, grouped in 4 blocks of 25 trials; blocks were separated by a resting pause of a few minutes.

In the *explicit* condition subjects were instructed to pay attention to the sinusoidal hand movement of the mover’s hand resting on the metal platform. In both *semi-implicit* and *implicit* conditions a small LED light was fixed on the dorsal surface of the second phalanx of the middle finger of the mover’s hand. In each trial of 10 hand oscillation cycles both frequency and number of LED flashes (duration of the light flash 200ms) varied randomly; the maximal on/off frequency was 2Hz, so that each LED flash could be clearly separated perceptually. Therefore, during each 10s trial the LED could flash from a minimum of 1 time to a maximum of 20 times, both frequency and number varying unpredictably. The LED sequence was produced in each single trial by an *ad hoc* random signal-generating program. Hand movement and onset of the LED flash series were synchronized at the beginning and proceeded independently, so that during the 10s period the LED could flash at any time during the hand oscillation cycles. A beeping sound signaled both the beginning and the end of each 10s trial. In the *semi-implicit* condition, subjects were instructed to pay attention to the LED light and report whether, when the LED light mounted on the mover’s hand had flashed for the last time in each 10s trial, the moving hand had been flexed upward or downward, or was in the intermediate, horizontal position. The task required constant attention because subjects did not know when the LED would flash for the last time. The subjects’ attention needed to be partly allocated to the moving hand, since they needed to monitor hand position in order to give the correct answer, but subjects were never explicitely instructed to pay attention to the hand movement. In the *implicit* condition, different subjects were instructed to pay attention to the LED light and to count and report the number of times the LED light had flashed during each 10s trial. Because of the unpredictability of the LED activity, the task required constant attention. Subjects were never instructed to pay attention to the hand movement, which in fact was irrelevant to their answer. In both *semi-implicit* and *implicit* experiments subjects received immediate feedback on the accuracy of their performance (at the end of each 10s trial). Finally, in the *baseline* condition subjects were instructed to pay attention to the sinusoidal movement of the metal platform. The platform was connected to the hand of a mover hidden behind a screen, by a long rod attached to its pivot, so as to produce an oscillating movement with the same kinematic characteristics as that observed during the flexion-extension of the mover’s hand.

## Data acquisition

H-reflexes were evoked in the Flexor Carpi Radialis (FCR) muscle by electrical stimulation of the Median nerve at the elbow (square pulse, 0.8 ms duration), and recorded with external bipolar electrodes placed on the muscle belly. Signals were amplified, filtered (10–1000 Hz) and A/D converted (5 kHz sampling rate). Peak to peak amplitude of the FCR H-reflex at rest was maintained between 5 to 15% of the maximal direct motor response (Mmax). In order to exclude the possibility of voluntary or involuntary muscular activity in the observing subjects, i.e. that, contrary to instructions, the flexor or extensor wrist muscles would actually be active during observation, the background EMG was continuously monitored in the FCR and in its antagonist *(Extensor Carpi Radialis)*, throughout the 10s of each movement observation trial. This actually never occurred, so that no trials were ever discarded. Different experimental conditions were tested in different subjects to avoid influencing the allocation of subjects’ attention based on previous experience, and because the registration of the H-reflex response in all conditions in a single subject would have led to response adaptation after prolonged stimulation of the peripheral nerve and would have been uncomfortable for the subject. All signals (H-reflex and movement traces) were recorded and stored for later analysis.

**Fig 1:**
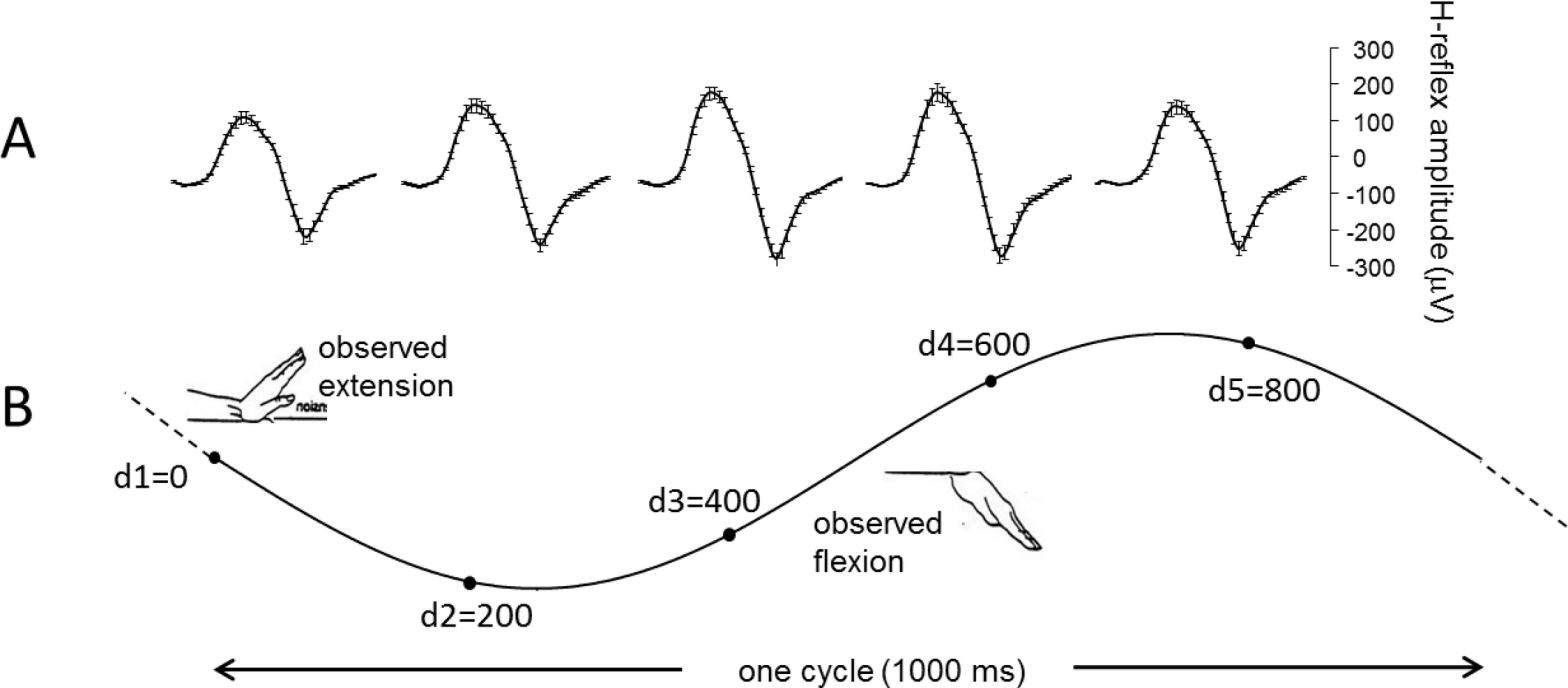
Data acquisition and experimental protocol. (A) Average traces (μV ±SEM) of 25 H-reflexes recorded from a single subject, in a single trial of movement observation in the *explicit* condition. (B) Average sinusoidal time course of 25 flexion-extension hand movements. Black dots on the sinewave indicate the 5 different delays during the hand flexion-extension cycle in which reflexes were recorded (d1 = 0, d2 = 200, d3 = 400, d4 = 600, d5 = 800 ms) corresponding to 5 different hand angular positions, dividing the 1Hz oscillation cycle in five equal parts. Note the motor resonant response, i.e. the modulation of the reflex amplitude matches the cyclic time course of the observed movement, with smaller reflexes recorded during observation of the extension phase (e.g. d1 and d5) and larger ones during the observation of the flexion phase (e.g. d3 and d4).

To describe the specific temporal relation between the time courses of excitability modulation in the FCR muscle and observed movement, H-reflexes were recorded at 5 different points in time during the hand flexion-extension cycle (0, 200, 400, 600, 800 ms) corresponding to 5 different hand angular positions, dividing the 1s oscillation cycle in five equal parts (Fig 1). In order to synchronize and to lock the timing of physiological responses in the observer and observed hand movement by the mover, platform (hand) position was used as a triggering signal for electrical stimulation and data acquisition. When, during the third hand oscillation the platform reached a pre-selected position in the cycle, a trigger signal was released to activate the stimulator to elicit an H-reflex in the FCR muscle of observers and data acquisition, at one of the 5 different delays. Therefore, H-reflex samples were always taken during the third of 10 hand oscillation cycles. Delays were selected automatically by the acquisition program in semi-random order, i.e. completing a cluster of all 5 delays before starting the new random selection again.

### H-reflex technique

This is a well-established neurophysiological technique, first described by Hoffmann in 1918 [61]. The stimulation of spindle afferences from a given muscle (here the FCR muscle, by electrical stimulation of the Median nerve) induces a monosynaptic activation of the spinal motorneurons innervating the homonymous muscle and, in turn, its contraction, i.e. the H-reflex response, measurable by EMG. The amplitude of the response is related to the number of motor units activated through the reflex arc, which in turn depends on stimulus intensity. When the number of the afferent fibers stimulated is kept constant, with a stimulus of constant intensity, the variation of the amplitude of the response, in the absence of movement, depends on motorneuron excitability modulation by descending cortical pathways. Specifically for this experimental setup, the amplitude modulation of the H-reflex during action observation has been shown to depend only on changes in cortical activity reaching spinal motorneurons via the M1 component of the corticospinal tract [47].

### Data analysis

In all conditions, averages of all recorded single hand movements were calculated and fitted by a four-parameter (period, offset, amplitude and phase) sinewave function. Parameters of the best-fit equation were calculated by minimizing the sum of the squared differences between the observed and predicted values of the hand angular position (Marquardt-Levenberg algorithm, SigmaPlot). These records were then normalized to their calculated average cycle period because, despite being paced by a metronome, the mover’s hand cycle period varied among trials by about 5% of its average value: thus normalization was necessary to bring movement records from different trials back to unity (1s). Maintaining the temporal lock between the time courses of observed movement and response modulation in observers was critical in these experiments, therefore in each subject the same temporal normalization was also performed on each of the 5 delays at which the H-reflexes were recorded and average reflex values obtained at the same delay were assigned to their corresponding normalized delay. In order to minimize sources of variability of H-reflex amplitude over time (each experimental session lasted about 60 min) and thus independent of the experimental manipulation, in each observer the deviation (in μV) from the mean of the 5 responses recorded in each cluster of delays was calculated for each delay. This last value was then averaged with those obtained at the same delay in the other clusters. Thanks to this procedure, average data points from all different subjects, in each experiment, could be plotted together and was then fitted with a common two parameters (amplitude and phase since, after normalization, period = 1 and offset = 0 for all) sinewave function. Significance of these sinewave regressions was ascertained with a standard analysis of variance.

Behavioural responses, i.e. number of errors made by subjects in the semi-implicit and implicit conditions were averaged within each condition and compared utilizing a Kruskal-Wallis one-way ANOVA.

A circular-linear correlation analysis was utilized to correlate the angular position of the oscillating hand with the amplitude of H-reflex modulation in all subjects, in the three hand-movement conditions (*explicit*, *semi-implicit* and *implicit* observation) and in the *baseline* condition (observation of metal platform). First, the significance of the circular linear correlation was calculated utilizing all subjects of each condition (n = 14), and for each subject the 5 average reflex amplitude values, recorded at the 5 given angular position of the oscillating hand, corresponding to the 5 delays at which H-reflexes were recorded. Then, to compare the goodness of the correlation for the different conditions, the single subject correlation coefficients were utilized, obtained from all data points in each subject. Single subject R values were Fisher transformed to obtain a normal distribution and then compared utilizing a one-way ANOVA, followed by LSD post hoc tests.

Differences in amplitude of the motor resonant responses in all conditions were evaluated by comparing the single subject H-reflex modulation amplitude parameter, derived from the sinewave function fitting each subject’s average data points, with a Kruskal-Wallis one-way ANOVA, followed by Mann-Whitney U post-hoc tests.

Phase differences between observed movement and resonant response in the *explicit*, *semi-implicit* and *implicit* conditions were derived from the sinewave function fitting each subject’s average data points. The key element in the comparison of this parameter in the different conditions was the variability within each group of subjects belonging to each condition (see Results). For this reason in each condition phase differences were normalized by dividing single subject values by the standard deviation of the mean of those values. Subsequently normalized data were compared with a one-way ANOVA, followed by Duncan’s post-hoc tests. Group phase differences between observed movement and resonant response in all 3 conditions were also calculated utilizing the sinewave function fitting the plot of cumulative data points from all subjects. Significance of these sinewave regressions was again ascertained with a standard analysis of variance.

Parametric and non-parametric tests were utilized in respect of standard statistical assumptions regarding data distribution and variance. For all statistical tests, significance level was set at p<0.05. Data were acquired and recorded using a custom program in LabView13 and stored for later analysis; statistical analysis was conducted using SigmaPlot or SPSS software (SPSS Inc, Chicago, USA). All relevant data are available as a Supporting Information files (S1 Table, S2 Table and S3 Table).

## Results

### Muscle selection and time course of the response

**Fig 2:**
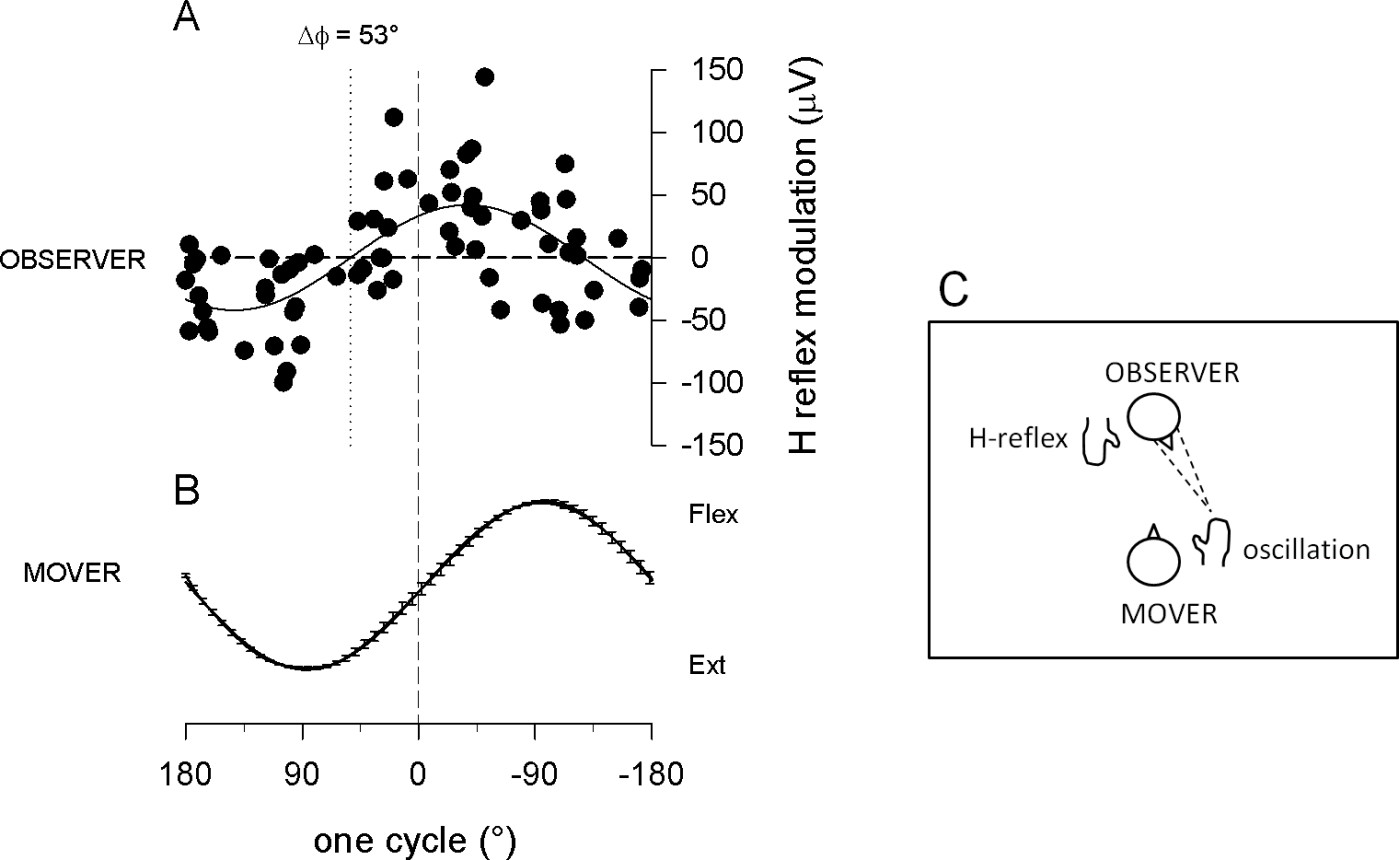
Explicit observation condition. (A) H-reflex amplitude modulation recorded in the right FCR muscle of 14 right-handed observers, during observation of one cycle of a flexion-extension movement of the mover’s right hand (B, average movement trace performed by the mover in all different experiments (±SEM)). In panel A the cumulative plot of the average data points from all subjects is fitted with a sinusoid equation with the same period as that fitting the movement. H-reflexes are significantly modulated, showing that increasing excitability of the flexor motorneuronal pool develops during the flexing phase of the observed movement. Δϕ: phase difference between reflex modulation in flexor muscle of the observer and hand oscillation of the mover. For easier graphic visualization of the parallel time course of the two events, hand flexion (Flex = downward direction of the moving hand) is drawn in the upper direction. (C) Graphic representation of this, and all following experiments.

To compare the effect of attention manipulation in the different experimental conditions, the first necessary step was to verify whether in all conditions the time course of FCR H-reflex amplitude modulation remained significantly correlated with the time course of the hand movement, as it is in the *explicit* observation condition (Fig 2). In this condition, H-reflexes recorded in the right FCR muscle of right-handed observers were significantly modulated during observation of the mover’s hand flexion-extension movement, with increasing excitability developing in the flexor motorneuronal pool during the flexing phase of the observed movement. Fig 2 (panel A) shows the cumulative plot of average data points from all subjects, aligned after time normalization and fitted with a common sinewave function (R^2^ = 0.42, p<0.0001), with the same period as that fitting the average movement (panel B; R^2^, = 0.99, p<0.0001).

**Fig 3:**
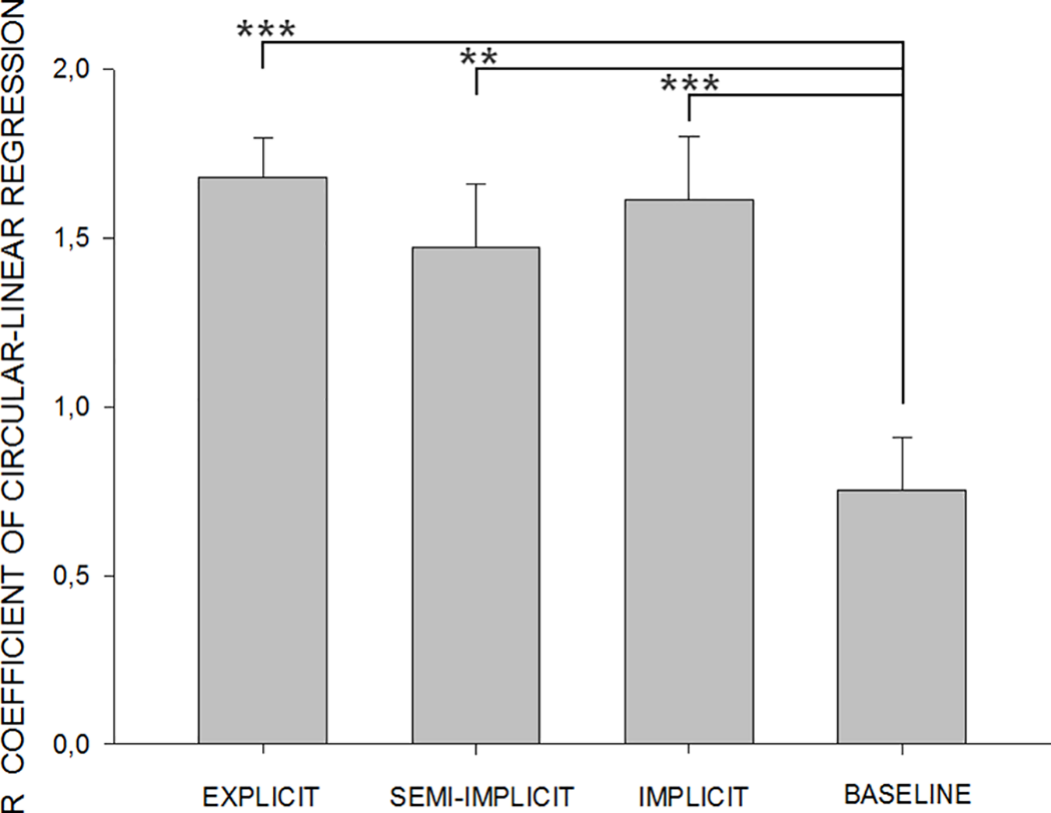
Sinusoidal time-course of reflex amplitude modulation. Average correlation coefficients of the circular-linear analysis (±SEM) obtained in the *explicit*, *semi-implicit* and *implicit* conditions are significantly different from the R coefficients obtained in the *baseline* condition (** p≤0.01, ***p≤0.001).

In the *semi-implicit* and *implicit* conditions, when the attention of subjects was not directed to the observation of the hand movement visible in front of them, but to the LED light events, a motor resonant response still developed, correctly linked to the time course of the movement (though with a dramatic reduction in the amplitude of the reflex modulation, see “Amplitude decrease” section). The results of all different attention conditions were analysed and compared to each other, as well as to those of the *baseline* condition, using a circular-linear correlation. The correlation resulted significant for all but the *baseline* condition (*explicit* observation R = 0.64, p = 0.006; *semi-implicit* observation R = 0.40, p = 0.004; *implicit* observation R = 0.42, p = 0.002), which, as expected, did not have a sinusoidal time course (circular-linear correlation: R = 0.07, p = 0.84). A one-way ANOVA was performed on Fischer transformed R coefficients obtained in the correlation analysis for each single subject (Fig 3), showing that the correlation coefficients of the circular-linear analysis were not different in the *explicit*, *semi-implicit* and *implicit* observation conditions, but significantly smaller in the *baseline* condition (one-way ANOVA, F_3,52_ = 6,753, p = 0.001; LSD post-hoc, *baseline* vs *explicit* p = 0.000, vs *semi-implicit* p = 0.003, vs *implicit* p = 0.001).

### Amplitude decrease

**Fig 4:**
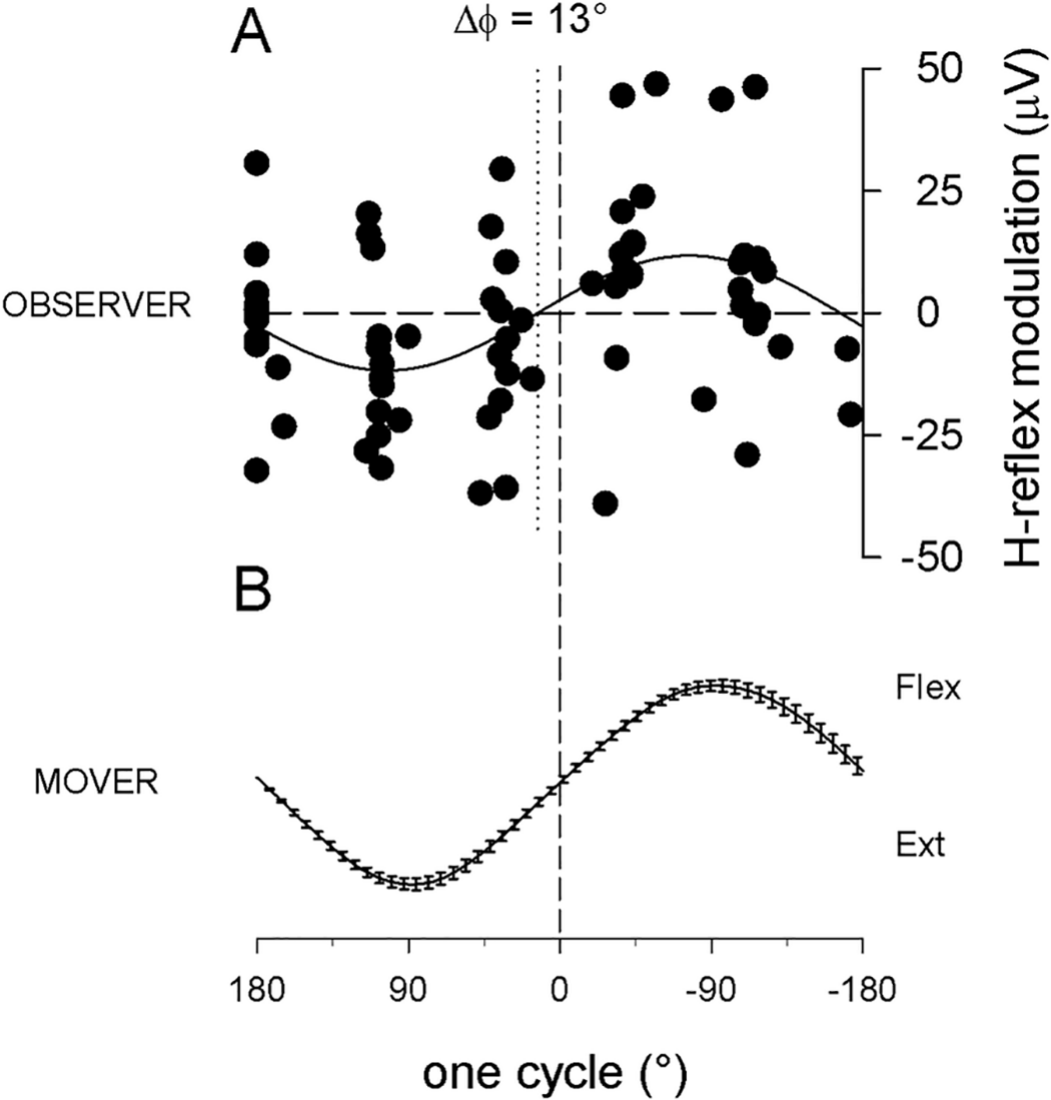
Semi-implicit observation condition. (A) H-reflex modulation recorded in the right FCR muscle of 14 right-handed observers, during observation of one cycle of a flexion-extension movement of the mover’s right hand (B, average movement trace performed by the mover in all different experiments (±SEM), when observers are explicitly instructed to report the mover’s hand position corresponding to the last time the LED light was flashed in each trial. In panel A the cumulative plot of the average data points from all subjects is fitted with a common sinusoid equation with the same period as that fitting the movement. Note the reduced scale of the ordinate compared to Fig 2A. Δϕ: phase difference between reflex modulation in flexor muscle of the observer and hand oscillation of the mover. Flex = downward direction of the moving hand.

In Fig 4 (panel A), the cumulative plot of average H-reflex amplitude data points from all subjects of the *semi-implicit* condition (tell hand position when the LED light was last flashed) aligned after time normalization, was fitted with a common sinewave function with the same period as that fitting the average movement (panel B; R^2^, = 0.99, p<0.0001), and sinusoidal H-reflex modulation was highly significant (R^2^ = 0.19, p<0.001). Note the reduced scale of the ordinate compared to Fig 2A. The task, reporting whether when the LED light flashed for the last time the moving hand was up, down or horizontal, required constant attention and subjects made very few errors (number of errors across all subjects: average = 3.13 (±1.98 SD) errors/block of 25 trials; mode = 3; min = 1, max = 8).

**Fig 5:**
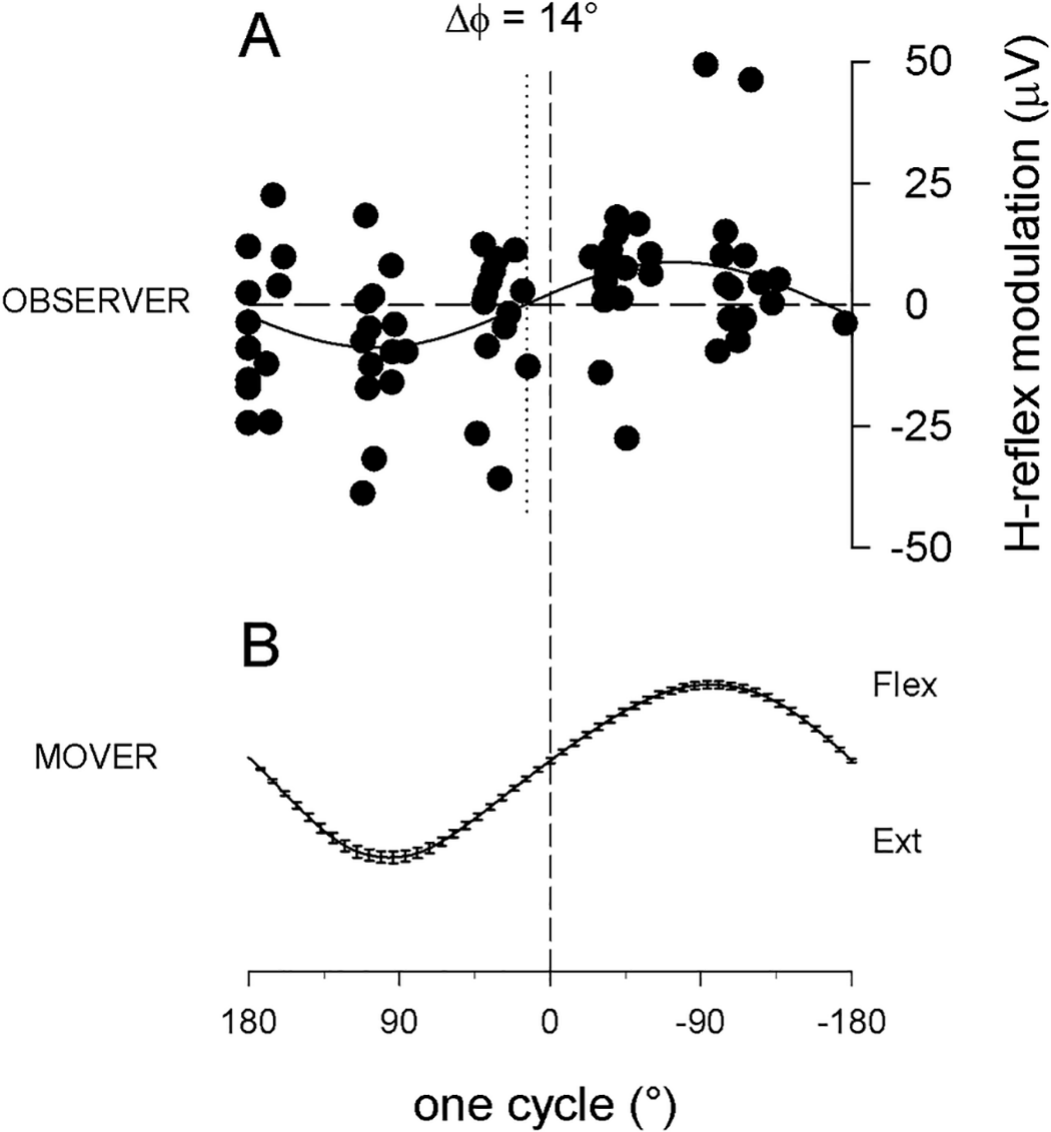
Implicit observation condition. (A) H-reflex modulation recorded in the right FCR muscle of 14 right-handed observers, during observation of one cycle of a flexion-extension movement of the mover’s right hand (B, average movement trace performed by the mover in all different experiments (±SEM)), when observers are instructed to count how many times in each trial the LED light on the moving hand was flashed. In panel A the cumulative plot of the average data points from all subjects is fitted with a common sinusoid equation with the same period as that fitting the average movement. Note the reduced scale of the ordinate compared to Fig 2A. Δϕ: phase difference between reflex modulation in flexor muscle of the observer and hand oscillation of the mover. Flex = downward direction of the moving hand.

In the *implicit* movement observation condition (Fig 5) subjects were instructed to count how many times the LED light flashed during each trial. In panel (A) of Fig 5 the cumulative plot of average data points from all subjects, aligned after time normalization, are fitted with a common sinewave function, with the same period as that fitting the average movement (panel B; R^2^, = 0.99, p<0.0001) and sinusoidal H-reflex modulation is highly significant (R^2^ = 0.17, p<0.002). Note the reduced scale of the ordinate compared to Fig 2A. This task also required constant attention and subjects made very few errors (number of errors across all subjects: average = 2.66 (±2.5 SD) errors/block of 25 trials; mode = 2; min = 0, max = 9). The number of errors in the *semi-implicit* and *implicit* conditions was not significantly different (Kruskal-Wallis one-way ANOVA, H(1) = 3.404, p = 0.065), indicating that the two tasks had similar difficulty.

**Fig 6:**
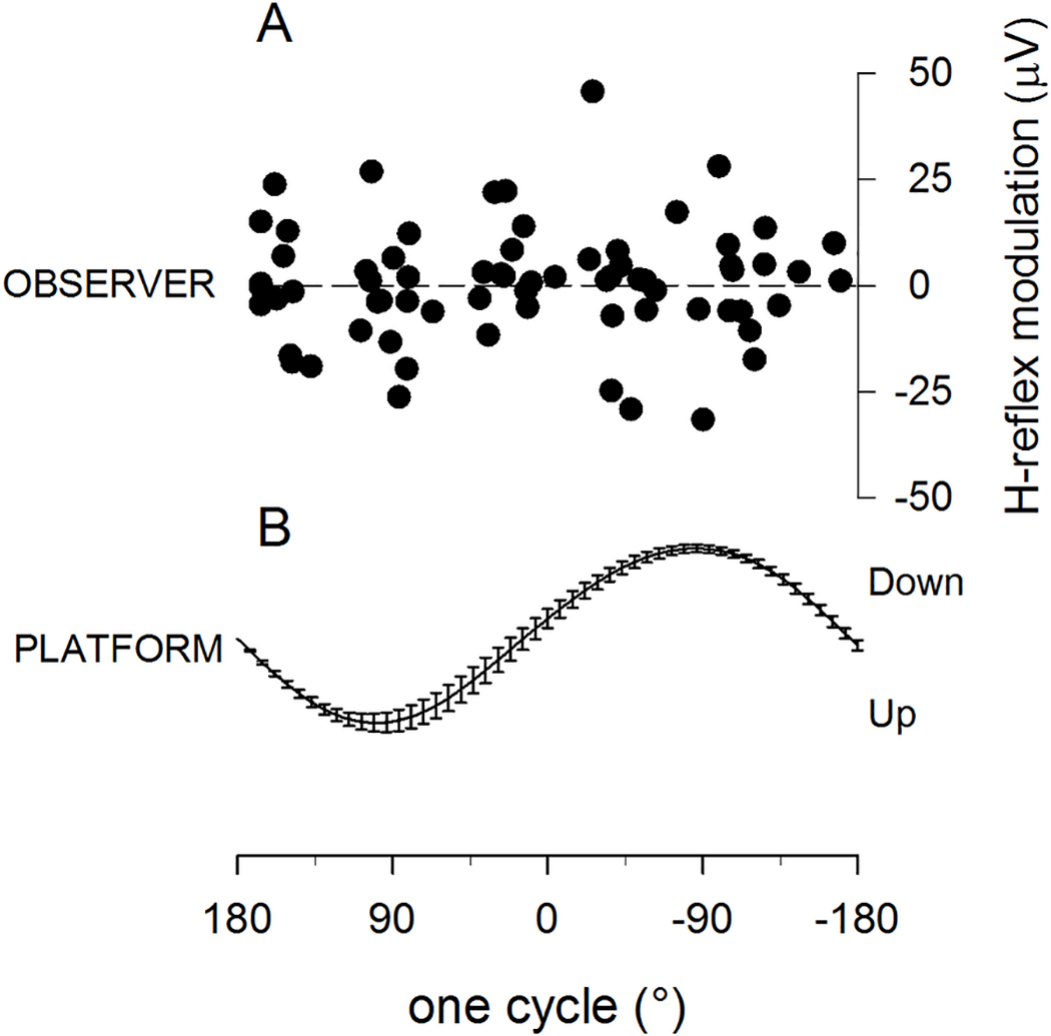
Baseline observation condition. (A) H-reflexes recorded in the right FCR muscle of right-handed observers are not modulated during observation of one cycle of a sinusoidal movement of a metal platform (B, average movement trace performed by the platform in all different experiments (±SEM). In panel (A) the cumulative plot of the average data points from all subjects could not be fitted by a sinewave. Down and Up = directions of the moving platform. No resonant response is recorded when subjects are observing the metal platform oscillating without the hand (with the same sinusoidal rhythm as in Figs 2, 4 and 5, and H-reflex amplitude variations are the result of random variability rather than a modulation induced by the observation task.

When subjects were instructed to observe a moving platform with no hand on it (*baseline* condition), the motor resonant response did not develop (Fig 6). The amplitude of FCR H-reflexes in this condition reflects random variations rather than being modulated with the time course of the observed movement of the metal platform (panel B; R^2^, = 0.99, p<0.0001). In panel (A) of Fig 6 the cumulative plot of the average data points from all subjects, aligned after time normalization, could not be fitted by a sinewave function with the same period of the observed movement (R^2^ = 0.0015, p = 1). This experiment replicates previous results (Borroni et al., 2005) showing that in order to induce a motor resonant response the oscillating movement must be executed by a hand, while a simple mechanical device is ineffective, and provides a baseline reference of random H-reflex variability for comparison with responses in the other experimental conditions.

**Fig 7:**
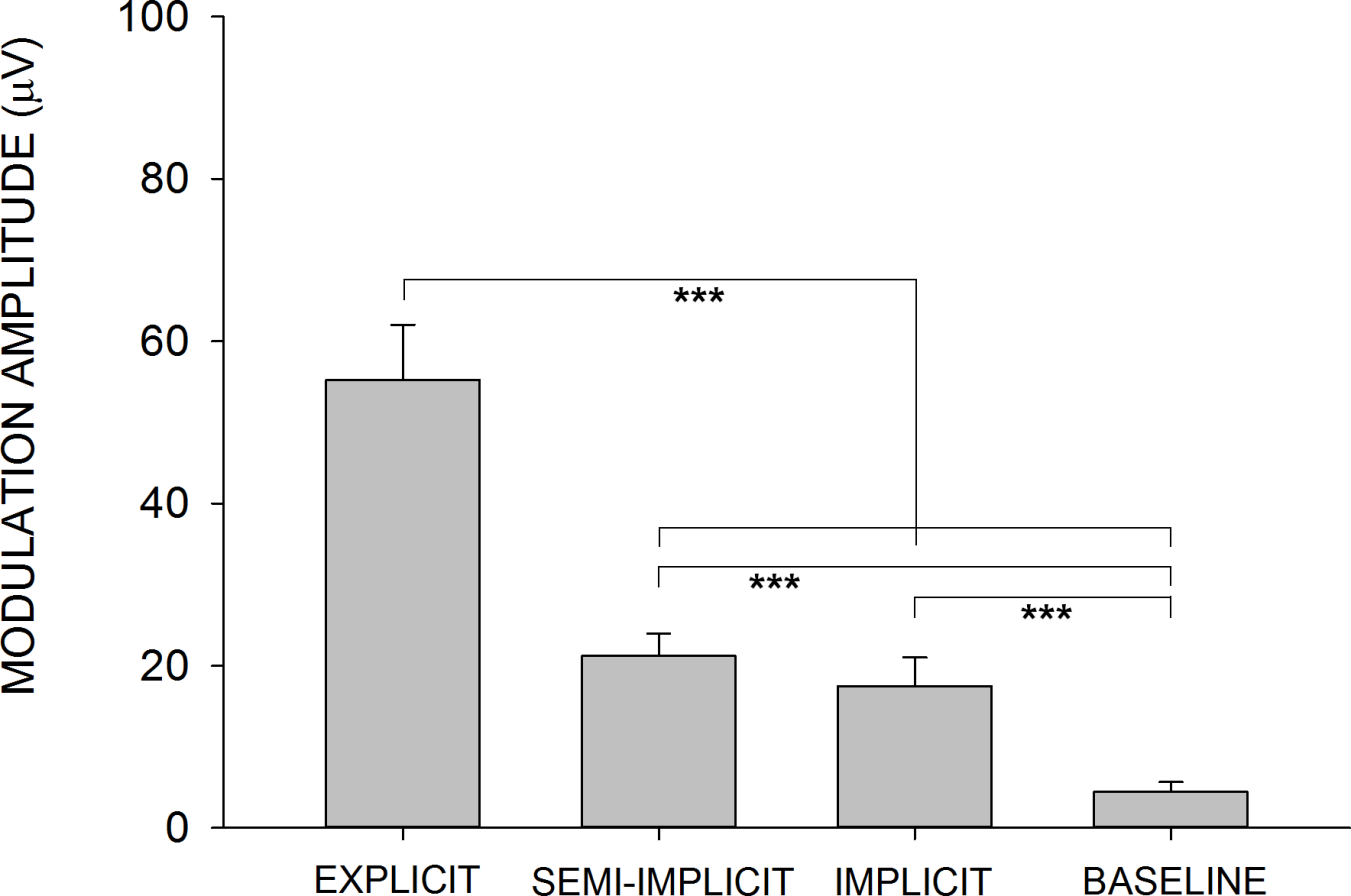
Amplitude of H-reflex modulation. Derived in each subject from the sinewave function fitting the subject’s average data points in the *explicit*, *semi-implicit* and *implicit* observation conditions, the average H-reflex amplitude modulation (± SEM) is significantly larger in the *explicit* condition compared to each of the other conditions, and is significantly smaller in the *baseline* condition compared to the *semi-implicit* and *implicit* conditions, which were not different from each other (***p<0.001).

To quantify the effect of directing attention to other tasks rather than to action observation, on the development of the motor resonant response, the amplitude of H-reflex modulation in the different experimental conditions was compared (Fig 7). The reflex modulation amplitude parameter was derived from the sinewave function fitting each subject’s average data points. In the *baseline* condition fitting were extremely poor, with several R^2^ values very close to zero; obviously this measurement of amplitude has no physiological meaning and the poor correlation confirms that in this condition motor resonance does not develop; nonetheless numerically it is a useful tool, precisely because it establishes a baseline experimental condition, with no resonant response in a very similar experimental protocol, against which to compare the other conditions. Fig 7 shows that the H-reflex amplitude modulation was different in the different observation conditions (Kruskal-Wallis one-way ANOVA, H(3) = 36.127, p = 0.000); it was significantly larger in the *explicit* observation condition compared to all other conditions (*explicit* vs. *semi-implicit* (p = 0.001), *explicit* vs. *implicit* (p = 0.000), and *explicit* vs. *baseline* (p = 0.000), and significantly larger in the *semi-implicit* and *implicit* conditions (not different from each other, (p = 0.15), compared to the baseline condition (*semi-implicit* vs. baseline p = 0.000); *implicit* vs. *baseline* (p = 0.000), Mann-Whitney U post-hoc tests, Bonferroni correction (p < 0.008).

### Phase unlock

**Fig 8:**
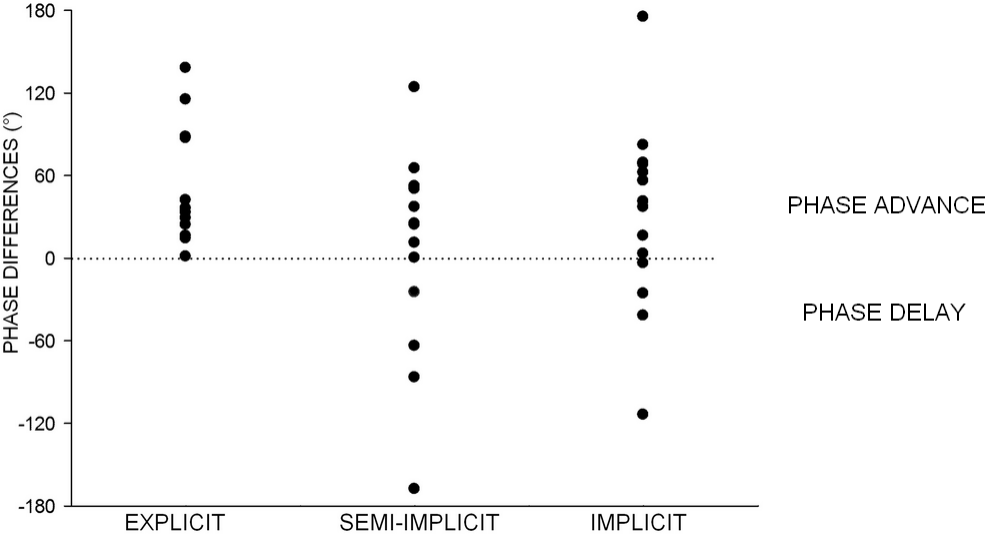
Phase differences between observed movement and H-reflex modulation. Derived in each subject from the sinewave function fitting the subject’s average data points in the *explicit*, *semi-implicit* and *implicit* observation conditions. Note that in the *explicit* condition phases are always in advance of the observed movement (as in the actual execution of the same movement), whereas in the *semi-implicit* and *implicit* conditions they are scattered across the entire possible range (-180° to +180°).

Finally, as evident from comparing Figs 2, 4 and 5, the phase relationship between the two sinusoidal functions fitting the H-reflex data and the hand movement describing when the resonant response occurs with respect to the observed movement (anticipating or following it), was also affected by a redirection of attentional resources normally dedicated to the observation of the action observation task. In line with previous studies performed with *explicit* observation, in Fig 2 the H-reflex modulation anticipates the movement with a phase difference of 53°, while in Figs 4 and 5 this phase advance is reduced to 13° and 14° respectively; the latter values, however, do not reflect a greater synchronization of the motor resonance response to the observed movement in the *semi-implicit* and the *implicit* conditions, which is only apparent. In order to explain this matter we must look at single subject responses. In fact, values obtained from the sinewave functions fitting each subject’s data points (Fig 8) range from 1 to 138 in the *explicit* condition and from -168 to 124 in the *semi-implicit* and from -114 to 175 in the *implicit* conditions. Therefore, phase differences in these two conditions are not at all consistent in all subjects (as they are in the explicit condition) but rather result from phase differences that vary within the entire possible range (-180° to +180°) and indicates that time course of hand movement and resonant motor program have become uncoupled. In order to capture the difference in distribution of phase values within each group of subjects belonging to each condition, phase differences were normalized by dividing single subject values by the standard deviation of the mean of those values. Subsequently, a one-way ANOVA performed on averaged normalized data showed that phase differences in the 3 conditions were significantly different (p = 0.01), with the *explicit* condition that was different from either the *semi-implicit* (p = 0.003), and *implicit* (p = 0.042) conditions, which were not different from each other (p = 0.3), as shown by post-hoc LSD tests.

## Discussion

This study investigates the role of attention in shaping motor resonant responses and reveals a new, multifaceted interaction, between deployment of attentional resources and resonant activation of motor circuits. The H-reflex technique was utilized to quantify the amplitude of responses and the muscular and temporal accuracy of the subliminal motor program (the facilitation of spinal motorneurons in the right muscle and at the right moment during the time course of the observed action), in an experiment with four different conditions: 1) *explicit* observation (Fig 2), in which subjects were asked to pay attention exclusively to a 1Hz cyclic oscillatory movement of a hand; 2) *semi-implicit* observation (Fig 4), in which subjects had to attend a different task requiring deployment of attentional resources and—albeit implicitly- the same hand movement, viewed in the background; 3) *implicit* observation (Fig 5), in which the hand movement viewed in the background was totally irrelevant to the completion of the task; and 4) a *baseline* reference condition.

Results are, first of all, identical in both *semi-implicit* and *implicit* observation conditions, indicating that the viewing of an action is, *per se*, a powerful stimulus, so that a motor resonant response develops even when the action is not the primary focus of subjects’ attention and even when it is irrelevant to the task. In these conditions a basic resonant activation of motor circuits, i.e. the essence of the subliminal resonant motor program for a hand oscillation at 1Hz, is evoked: modulation of the correct muscle and sinusoidal time course of the response. However, attention manipulation dramatically decreases the amplitude of the motor resonant response and uncouples the phase relation between observed movement and excitability modulation of motor pathways in observers. We conclude that the complete motor resonant response requires full attention to develop, but that different parameters of the response are differently affected by subtraction of attentional resources. The kinematic details of the response, such as amplitude and its optimal phase anticipation with respect to observed movement, require full attention, since coding for these movement parameters is disrupted when attentive resources are diverted towards another, primary, task. While, more general parameters of the motor resonant response, such as muscular selection and time course, appear to be able to automatically capture the necessary amount of attentive resources.

### Muscle selection and time course of the response

The correlation between the cyclic time course of FCR H-reflex amplitude modulation and the cyclic time course of the observed movement remained significant in the *implicit* and *semi-implicit* conditions (despite the overall decrease in amplitude) and, in fact, the specificity of this time course was not different from that recorded in the *explicit* observation condition (Fig 3). Since muscle selection and temporal specificity of the motor resonant response are correctly activated, this response could be viewed as the subliminal motor program representing the observed 1Hz hand oscillation movement, which retains all the essential elements of the observed action, i.e. the period of the wrist flexion-extension and the activation of the flexor muscle in the correct half of this period. When executed, the hand oscillation movement is driven by antagonist muscle groups (flexors and extensors) and FCR is one of the primary wrist flexors. In the explicit observation condition (as during execution) the H-reflex in the FCR is facilitated not at any time during the flexion-extension movement, but at the correct time during the observation of the hand flexion (Fig 2). It has previously been shown that, in the same observation condition, TMS-induced Motor Evoked Potentials in the antagonist muscle (ECR, not recorded in the present experiment) are also facilitated not at any time during the flexion-extension movement, but at the correct time during the observation of the hand extension[46]. Because the fitting of the reflex data with a sinusoidal function with the same period as the observed movement is highly significant, we can conclude that both observed movement and reflex modulation have the same sinusoidal time course, i.e. that he FCR pattern of activation varies continuously (even though for practical reasons we only sample it at fixed intervals) with the observed movement.

We interpret this result as evidence in favor of the idea that biological movement is a powerful stimulus for the AON, which is recruited to assemble the general motor program associated with the motion viewed in the background while the subjects’ attention is focused on a different task. This response could perhaps be considered the core of the motor representation of the observed movement, deriving from an automatic recruitment of the AON, and providing the necessary and sufficient information to achieve a direct “motor” recognition of the observed action, as originally proposed by the direct-matching hypothesis [3,62]. On the contrary, the full-fledged activation of the corticospinal pathway measured in the *explicit* observation condition, which has all the motor details encoding the specific kinematics of the observed movement, is consistent with a more direct function of the resonant response in imitation and motor learning, for which kinematic details are essential. Further experiments using more challenging cognitive manipulations are necessary to verify whether it is possible to cancel even this core motor resonant response, by subtracting more/all attentive resources from the observation of the action.

### Amplitude decrease

When a cognitive task was performed while viewing the hand movement, i.e. when subjects were not instructed to pay explicit and focused attention to the movement in the *implicit* and *semi-implicit* conditions, the amplitude of the motor resonant response was profoundly affected and was subject to a dramatic decrease compared to the *explicit* observation condition (Fig 7). As a consequence of the decrease in attentional resources available for the coding of the movement, a reduced descending cortical command recruited a smaller number of spinal motorneurons. This result, seen from the opposite perspective, suggests that when full attention is devoted to an action observation task, the resulting motor resonant response is greatly amplified. Indeed, attention-dependent gain modulation of sensory processes is a well-described finding in sensory systems [63,64]. Generally, action observation studies have been carried out in conditions in which subjects were allowed or even required to observe the action with total attention. However, from a more naturalistic point of view, this is not the most common circumstance; on the contrary, in our daily life we are exposed to several simultaneous actions and perceptual events, with different meaning and consequences and not all can be equally relevant or interesting to the observer. Our data show that when attention is allocated to other tasks, actions remain very effective stimuli, capable of evoking motor resonant responses, but that the response is much reduced in amplitude and specificity.

Behavioral experiments have shown that the activation of motor responses during action observation, described as “automatic imitation”, in reality requires attention [65] and that if attention is so strongly diverted from the task that no cognitive resources remain available to process the observed action, the automatic imitation effect disappears [30]. Conversely, the effect is maintained, though reduced, when attentive resources are not exhausted [30]. On the other hand, several experiments have shown that observed actions automatically activate motor representations normally involved in the execution of those actions, even when subjects are performing other tasks [21,24,66–69]. According to the “load theory of attention” [70,71], when the completion of a primary task does not exhaust available attentional resources, irrelevant stimuli are also inevitably processed. In our experimental conditions, the instructions given to subjects were finalized to shift the focus of their attention from action observation to the LED light task (primary task). This task indeed absorbed some of the subjects’ attention, since they performed it correctly most of the time, making few errors, but the attentional capacity was probably not exhausted by the task, so that hand movement was also coded. However, the different components of the motor resonant response resulted differently susceptible to the availability of attentional resources and, in particular, response amplitude was strongly decreased, indicating that this component of the response requires that attention is focused only on the observed movement. Interestingly, the residual amplitude modulation observed in the present study is consistent with fMRI studies showing a residual activity in the AON when a secondary task or cognitive manipulation is imposed on subjects during action observation [72], although in that case it is not possible to say whether the decrease in BOLD signal has a functional correspondence in the modulation of the motor system. Our study suggests that this is the case, i.e. that redirecting attentional resources results in a reduction of the gain of neural processes leading to the subliminal activation of motor circuits, while preserving the overall shape of the resonant response. Similar results have also been described in experiments imposing cognitive manipulation on subjects executing—not observing—a movement. For example if subjects are distracted from their actions, it is more likely that they will make mistakes or perform the action more slowly [73]. Consistently, Johansen-Berg et al. (2002) [74] showed that reducing attention to finger movement by asking subjects to perform a concurrent counting task is associated with decreased BOLD signal in motor cortical regions, compared to the signal evoked by performing the movement without distraction.

### Phase unlock

Finally, the basic motor program recorded in the *semi-implicit* and *implicit* conditions, while maintaining a correct muscle selection and temporal specificity, is not useful to actually reproduce or imitate on-line the observed movement, given that the key element linking muscle contraction with resulting movement (phase advance) is lacking. This phase relationship, and factors affecting it, has been investigated in detail within the framework of motor execution [58]. The average phase difference measured in the *explicit* observation condition (Δϕ = 53°) reflects the average natural advance of the FCR muscle contraction with respect to the deriving hand flexion, in an executed 1Hz oscillatory movement with a prone hand [75]. During action execution such phase advance can change when mechanical parameters are modified as, for example, when the oscillatory movement at 1Hz is performed with a supine hand; importantly, also during the observation of the supine hand oscillation the phase advance changes accordingly [46]. In the present study, this phase relationship is completely lost in the *semi-implicit* and *implicit* conditions. The apparent decrease in phase advance (13° and 14° respectively, Figs 4 and 5) does not reflect a better synchronization between resonant response and observed movement, consistent in all subjects, but the average between single subject phase differences that vary practically over the entire possible range (-180° to +180°). While maintaining a sinusoidal time course, which well represents the time course of the movement, the resonant motor program has lost the functional features of a direct, time-locked subliminal on-line replication. The phase of the H-reflex modulation sine function is not always in advance; in some in some subjects it anticipates, while in others it follows, and in others yet it is in full anti-phase with the sine function of the observed movement. In summary, diverting attention from action observation generates resonant responses lacking key kinematic information. For this reason, we suggest that in these conditions only a more abstract representation of the observed movement survives, reproducing a generic hand oscillation, rather than the motor program specifically coding for the observed movement normally developing in full-attention action observation.

## Conclusions

The modulation of H-reflex during action observation tasks results from the modulation of activity in M1; to explain the reduction in amplitude of the modulation of motor pathways in the *semi-implicit* and *implicit* experimental conditions we hypothesize that M1 must receive less input from the rest of the AON (through premotor cortex), resulting in a reduced activation of corticospinal motorneurons and thus in a decreased amplitude modulation of the FCR H-reflex. What has emerged from the work of past few years, is that action observation does not automatically produce a full activation of motor pathways. The original contribution of the present study is the demonstration that while biological movement appears to be able to activate some portion of the AON even when attentional resources are not directly allocated to its observation, the resulting activation of motor circuits results much dampened and loses kinematic specificity. In this case the motor resonant response may still inform an automatic basic representation of the essential properties of the action, while explicit attention is necessary for a detailed representation of the kinematic parameters of the observed movement. Motor resonance therefore is not a uniform response, with unchanging properties and single function, but rather a composite phenomenon with different components [76] that are differently susceptible to cognitive manipulation [77] and may constitute parallel motor representations of the same observed action, utilized for different purposes by the central nervous system.

## Supporting information

S1 Fig Photograph of the experimental setup. (TIF)

S1 Table Dataset.reflex modulation_circular-linear analysis. (XLSX)

S2 Table Dataset.phase differences analysis. (XLSX)

S3 Table Dataset.amplitude analysis. (XLSX)
