# Supplementary figures and images for: The role of attention in human motor resonance
Source: PLoS One. 2017 May 16;12(5):e0177457. doi: 10.1371/journal.pone.0177457 (PMC5433684; doi:10.1371/journal.pone.0177457)

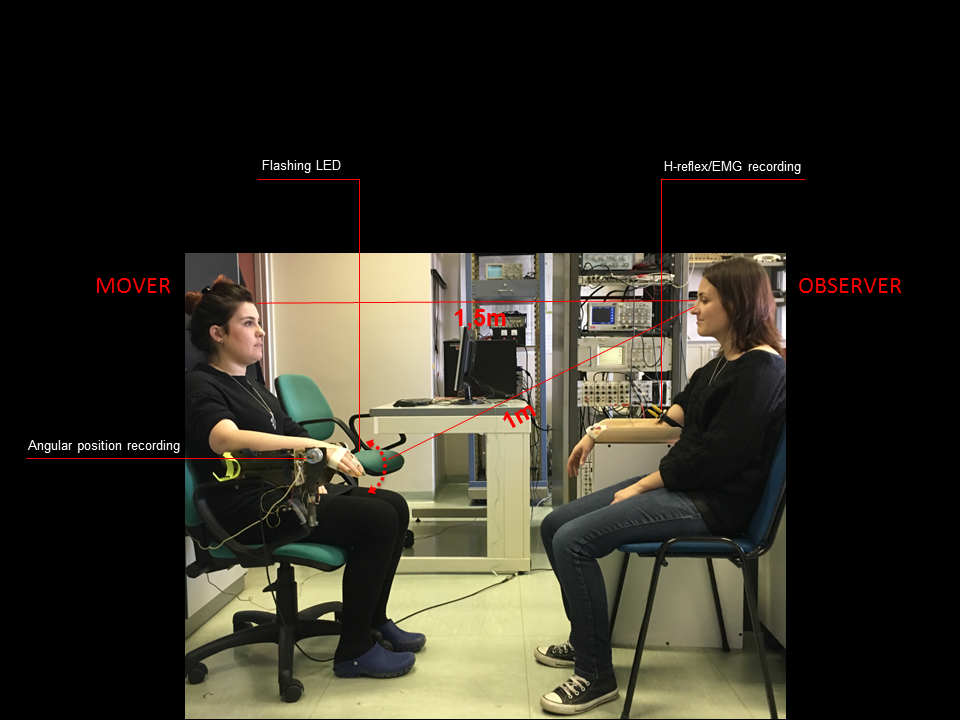

Supplement: S1 Fig — (TIF) [file pone.0177457.s001.tif]
